# *Sorbus domestica* Leaf Extracts and Their Activity Markers: Antioxidant Potential and Synergy Effects in Scavenging Assays of Multiple Oxidants

**DOI:** 10.3390/molecules24122289

**Published:** 2019-06-20

**Authors:** Magdalena Rutkowska, Monika Anna Olszewska, Joanna Kolodziejczyk-Czepas, Pawel Nowak, Aleksandra Owczarek

**Affiliations:** 1 Department of Pharmacognosy, Faculty of Pharmacy, Medical University of Lodz, ul. Muszynskiego 1, 90-151 Lodz, Poland; monika.olszewska@umed.lodz.pl (M.A.O.); aleksandra.owczarek@umed.lodz.pl (A.O.); 2 Department of General Biochemistry, Faculty of Biology and Environmental Protection, University of Lodz, ul. Pomorska 141/143, 90-236 Lodz, Poland; joanna.kolodziejczyk@biol.uni.lodz.pl (J.K.-C.); pawel.nowak@biol.uni.lodz.pl (P.N.)

**Keywords:** *Sorbus domestica*, oxidative stress, free radicals, reactive oxygen species (ROS), reactive nitrogen species (RNS), polyphenols, synergy

## Abstract

*Sorbus domestica* leaves are a traditionally used herbal medicine recommended for the treatment of oxidative stress-related diseases. Dry leaf extracts (standardized by LC-MS/MS and LC-PDA) and nine model activity markers (polyphenols), were tested in scavenging assays towards six in vivo-relevant oxidants (O_2_^•−^, OH^•^, NO^•^, H_2_O_2_, ONOO^−^, HClO). Ascorbic acid (AA) and Trolox (TX) were used as positive standards. The most active extracts were the diethyl ether and ethyl acetate fractions with activities in the range of 3.61–20.03 µmol AA equivalents/mg, depending on the assay. Among the model compounds, flavonoids were especially effective in OH^•^ scavenging, while flavan-3-ols were superior in O_2_^•−^ quenching. The most active constituents were quercetin, (−)-epicatechin, procyanidins B2 and C1 (3.94–24.16 µmol AA/mg), but considering their content in the extracts, isoquercitrin, (−)-epicatechin and chlorogenic acid were indicated as having the greatest influence on extract activity. The analysis of the synergistic effects between those three compounds in an O_2_^•−^ scavenging assay demonstrated that the combination of chlorogenic acid and isoquercitrin exerts the greatest influence. The results indicate that the extracts possess a strong and broad spectrum of antioxidant capacity and that their complex composition plays a key role, with various constituents acting complementarily and synergistically.

## 1. Introduction

**Figure 1:**
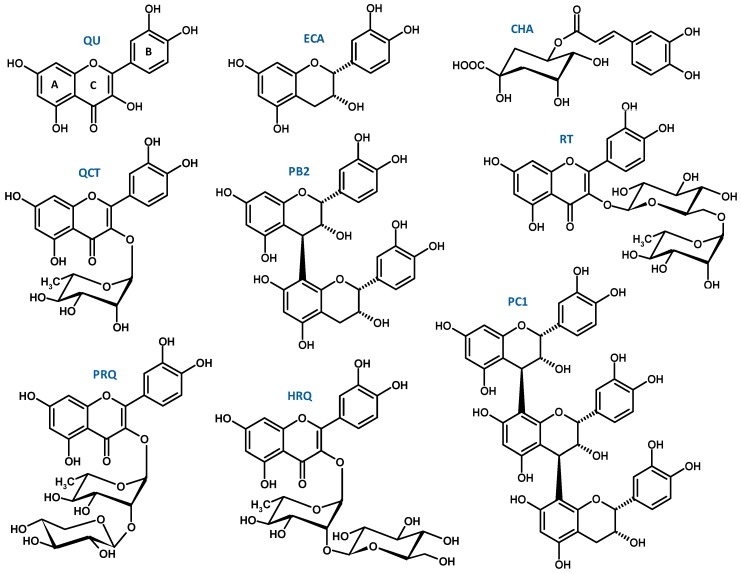
Structures of the activity markers of *S. domestica* leaves: ECA, (−)-epicatechin; CHA, chlorogenic acid (5-*O*-caffeoylquinic acid); PB2, procyanidin B2; PC1, procyanidin C1; QU, quercetin; QCT, quercitrin (quercetin 3-*O*-α-l-rhamnopyranoside); RT, rutin; HRQ, quercetin 3-*O*-(2″-*O*-β-d-glucopyranosyl)-α-l-rhamnopyranoside (HRQ); PRQ, quercetin 3-*O*-(2″-*O*-β-d-xylopyranosyl)-α-l-rhamnopyranoside; the capital letters A-C on the QU formula refer to the common nomenclature of the basic flavan skeleton.

*Sorbus domestica* L. is a wild rosaceous tree, native to the Mediterranean Basin, and cultivated as a dietary, medicinal, and decorative plant [1,2]. The polyphenol-rich leaves of this species are traditional herbal medicines. They are valued for their diuretic, antioxidant, anti-inflammatory, anti-atherogenic, and anti-diabetic properties and have been indicated in the treatment of prostatitis, nephritis, diabetes and hypercholesterolemia, among others [1]. Our ongoing studies of the molecular mechanisms of the leaves’ activity primarily focus on their influence on oxidative stress and inflammation—the two intertwined processes that have been found to play a crucial role in the development of numerous inflammatory and metabolic disorders, including those described in ethnopharmacological sources. In our previous work, we demonstrated that the leaf extracts inhibit pro-inflammatory enzymes (lipooxygenase and hyaluronidase), display protective effects against nitrative and oxidative damage to human plasma components (lipids and proteins) under oxidative stress conditions, and enhance the total non-enzymatic antioxidant status of plasma (NEAC) [3]. A detailed phytochemical study (LC-MS) of the leaf’s polyphenolic fraction revealed the occurrence of 44 individual compounds. Nine of them (Figure 1) were indicated as key markers of the extracts’ activity, based on their relative contribution to the total polyphenolic content (up to 74% depending on the extract) and their activity parameters in plasma [3,4].

Polyphenols are specialized (secondary) plant metabolites, believed to bestow beneficial effects on human health by their ability to counter the pathological consequences of oxidative stress [5,6]. The latter is a complex process involving multiple factors and mechanisms. However, its root cause is an excess of reactive oxygen/nitrogen species (ROS/RNS), which leads to unregulated oxidation of biological molecules, ROS/RNS-induced inflammation, and/or metabolic dysfunction [6,7]. Thus, one of the possible and most direct mechanisms of the protective effects of polyphenols and polyphenol-rich extracts against oxidative stress, and one that has not been studied so far for *S. domestica* leaves, is ROS/RNS quenching [6]. In biological systems, oxidative stress is generated by low-molecular weight ROS/RNS, such as superoxide (O_2_^•−^), hydroxyl radicals (OH^•^), hydrogen peroxide (H_2_O_2_), peroxynitrite (ONOO^–^), nitric oxide (NO^•^), and hypochlorous acid (HClO), varying in reduction potential, reaction mechanism, and selectivity towards biological molecules [8,9]. Effective protection from oxidative stress requires, therefore, antioxidants that are able to prevent different oxidative factors [8]. Due to their complex composition, plant extracts are likely to exhibit a broad reactivity and eliminate numerous ROS/RNS [6,10]. Additionally, the presence of multiple constituents raises the possibility of some synergistic effects [11]. Indeed, in the previous research, the activity of *S. domestica* leaf extracts was higher than what might be expected based only on the content of individual compounds [3,4]. However, no systematic evaluation of synergy for *S. domestica* leaves has been conducted so far.

Therefore, the objective of the present work, is to provide a clearer insight into the antioxidant potential of *S. domestica* leaves and its constituents by evaluating their scavenging potential towards multiple ROS/RNS of physiological importance, i.e., O_2_^•−^, OH^•^, H_2_O_2_, ONOO^–^, NO^•^ and HClO. Moreover, the synergistic effects between the representatives of the three main groups of *S. domestica* polyphenols (flavonols, flavanols and caffeoylquinic acid pseudodepsides) were tested in a selected model of O_2_^•−^ quenching.

## 2. Results and Discussion

### 2.1. Scavenging of Multiple Oxidants

The primary reactive species in human cells is O_2_^•−^, which is constantly generated during mitochondrial electron-transport chain, as well as via other endogenous reactions, e.g., those involving lipoxygenases, cyclooxygenases, xanthine oxidases, NADPH oxidases, or NO synthases. It also plays an important role in the generation of other oxidizing agents, including H_2_O_2_, OH^•^, HClO, and ONOO^–^ [8,9]. For instance, through the activity of superoxide dismutase (SOD), O_2_^•−^ is converted to H_2_O_2_. The latter is a rather stable form of ROS that, nevertheless, might be further converted to extremely reactive OH^•^ in the presence of various in vivo reductants [9]. Other non-selective and highly destructive oxidants include HClO, formed via the myeloperoxidase-catalyzed transformation of H_2_O_2_, and ONOO^‒^, produced in the reaction between O_2_^•−^ and NO^•^ [12,13]. Interestingly, only NO^•^ generated by inducible NO synthase (iNOS) in stimulated immune cells is a causative factor of ONOO^‒^ synthesis [14,15], whereas NO^•^ formed by endothelial NO synthase (eNOS) in the vessel endothelium is a beneficial antiatherogenic agent [12,15].

**Figure 2:**
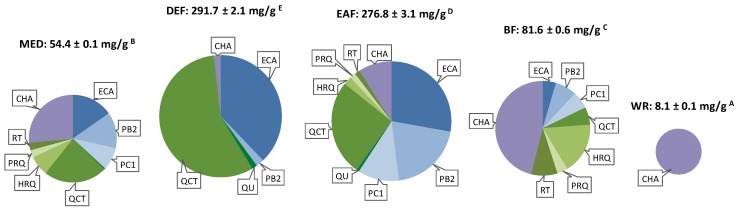
Total contents per g of dry weight of the *S. domestica* leaf extracts (LC-PDA-peaks; means ± SD, *n* = 3) and the relative proportions of the activity markers according to Matczak et al. [3]. The different letters A–E indicate significant differences (*p* < 0.05). MED, methanol-water (7:3, *v*/*v*) extract; DEF, diethyl ether fraction; EAF, ethyl acetate fraction; BF, *n*-butanol fraction; WR, water residue. For compound codes and structures, see Figure 1.

The analytes investigated in the present study were dry extracts from *S. domestica* leaves that have demonstrated effectiveness against ONOO^−^–generated oxidative damage of human plasma components, as well as the nine polyphenols selected previously [3,4] as the markers of the extracts activity (Figure 1). The fractionated dry extracts, i.e., the hydro-methanolic (7:3, *v*/*v*) extract (MED) and its fractions of different polarity (diethyl ether fraction, DEF; ethyl acetate fraction, EAF; *n*-butanol fraction, BF; and water residue, WR) were obtained and thoroughly standardized previously (LC-MS/MS, LC-PDA, and spectrophotometric profiling) [3]. The extracts varied in terms of total polyphenolic content and the total content and profile of the indicated markers (Figure 2), thereby comprising a good model for evaluating the contribution of individual components to the ROS/RNS scavenging potential of the leaves, as well as for screening for potential synergy effects.

**Figure 3:**
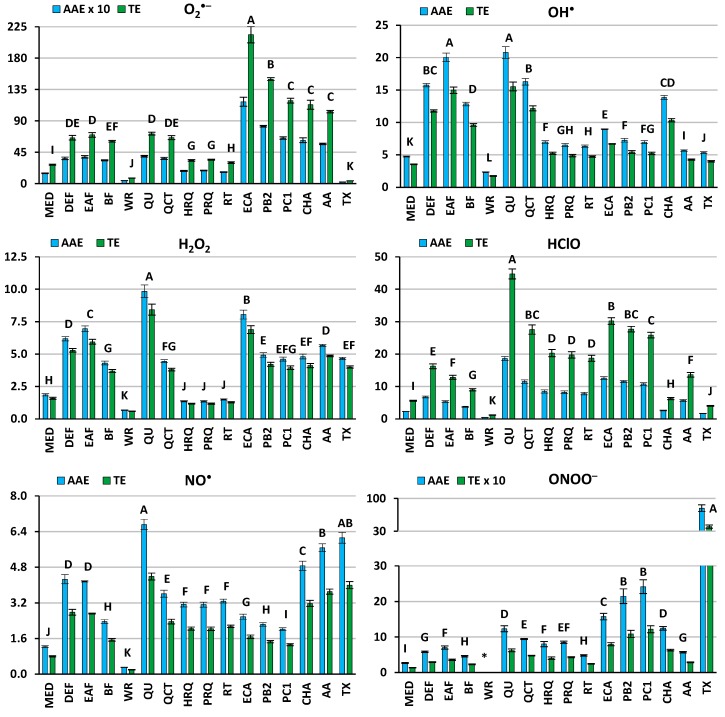
Antioxidant activity of *S. domestica* leaf extracts, their activity markers and standards towards in vivo-relevant oxidants. Results are expressed in micromolar equivalents of ascorbic acid (AA) and Trolox (TX) per mg of extract/compound and presented as mean values ± SD (*n* = 3). For each assay, the different letters A–K indicate significant differences (*p* < 0.05). The analyte marked with an asterisk was inactive in concentrations up to 200 µg/mL. For extracts and analyte codes, see Figure 1 and Figure 2.

To enable the direct comparison of the analytes’ capacities towards different ROS/RNS, the primary results (effective scavenging concentrations, SC_50_, Figure S1) were expressed as equivalents of positive standards—ascorbic acid (AA), the main water-soluble antioxidant of human plasma in vivo, and Trolox (TX), a synthetic analogue of vitamin E, which is the primary lipophilic (lipoprotein-bound) plasma antioxidant (Figure 3). Additionally, total activity scores AAE_MR6_ and TE_MR6_ for each analyte were calculated, understood as the sums of the analyte activities in all assays expressed in equivalents of AA and TX, respectively (Table 1).

All of the tested extracts showed a concentration-dependent ability to scavenge all of the target ROS/RNS (Figure 3), with total activity scores varying widely: 4.2–47.4 µmol AA/mg dw and 11.6–106.7 µmol TX/mg dw (Table 1). The highest AAE_MR6_ and TE_MR6_ were obtained for EAF and DEF, and then for BF, i.e., the fractions highly enriched in polyphenols (Figure 2). These findings are consistent with those obtained previously from simple chemical tests (TBARS, DPPH, FRAP) and from a model of human plasma exposed to oxidative stress [3]. The extracts were especially effective in OH^•^ scavenging, in which the most active fractions were 3–4 times more efficient than both of the standards (Figure 3). This observation may be of some physiological importance, as OH^•^ is a highly reactive, unselective radical able to oxidize almost every cellular component [9]. Other harmful species are also HClO and ONOO^‒^. Both are strong oxidants and additionally may cause chlorination and nitration disrupting the structure and function of proteins and other macromolecules [14,16]. Contrary to OH^•^, which reacts almost instantly at the place of origin, they can diffuse through membranes, causing a wider spread of oxidative and inflammatory processes [14,16]. Moreover, as HClO is not subjected to any endogenous antioxidant enzyme system, the low molecular-weight plasma antioxidants, both endo- and exogenous, become the primary line of defense [16]. In the case of both species, the most active fractions displayed comparable or higher activity to that of AA (Figure 3), and with regard to HClO, their efficiency was visibly superior to TX (Figure 3), suggesting that the extracts’ constituents may be able to support the antioxidants present in the biological system. In comparison to TX, the extracts were also highly effective towards O_2_^•−^. Despite not being very reactive, this species can still disrupt homeostasis, e.g., by targeting Fe-S centers of proteins and causing cluster disruption [17]. Moreover, scavenging of O_2_^•−^ may prevent the generation of the derived more harmful ROS/RNS. Only in the NO^•^-scavenging assay were the capacities of the extracts weaker (*p* < 0.05) than those of both reference antioxidants. Since NO^•^ has a positive function in intercellular signaling, vessel dilatation, the inhibition of inflammatory cell adhesion, and the promotion of fibrinolysis, targeting ONOO^−^ and O_2_^•−^, which gives ONOO^−^ a reaction with NO^•^, might actually be more beneficial [7,12,15].

Generally, the results suggest that the most active extracts obtained from *S. domestica* leaves may be considered broad-spectrum, strong antioxidants. Their scavenging activity may have positive effects in biological systems and, e.g., it might have been one of the protective mechanisms observed in the plasma model under the conditions of oxidative stress induced by ONOO^−^ [3].

The analysis of the quenching capacities of the *S. domestica* model constituents indicates their influence on the antioxidant capacity of the extracts. All of the tested compounds scavenged ROS/RNS dose-dependently (Figure 3), with total activity scores in the range of 25.4–72.2 µmol AA/mg (15.49–47.69 mol AA/mol) and 57.2–253.3 µmol TX/mg (34.91–135.58 mol TX/mol) (Table 1). The potential of the investigated polyphenols is especially apparent when compared in molar units (mol/mol) to the corresponding AAE_MR6_ of AA (6 mol AA/mol) and TE_MR6_ of TX (6 mol TX/mol).

Among the flavonoids, the highest activity parameters in all tests were observed for quercetin (QU) (Figure 3). It was also the most active analyte among the tested compounds, with regard to its AAE_MR6_ value (Table 1). Its glycosidic derivatives were less active, which is consistent with the common observation that glycosylation of the hydroxyl group in the C3 position lowers the antioxidant potential of flavonoids [18,19]. However, when the total antioxidant scores were expressed in mol/mol, the differences were much less pronounced and in the case of quercitrin (QCT), the AAE_MR6_ and TE_MR6_ values were equal to and higher than that of the QU, respectively (Table 1). This suggests that the lower activity of glycosides (when expressed in units of weight) is mainly due to their higher molecular mass, and that the free hydroxyl group at C3 position plays a less important role in quenching of the tested ROS/RNS than the catechol moiety and phenol groups in ring A of a flavonoid skeleton (Figure 1). Consequently, taking into account the content of the flavonoids in the extracts (Figure 2), QCT should be considered as having the greatest influence on the extracts’ activity.

The investigated flavan-3-ol derivatives gave the highest TE_MR6_ values, and their AAE_MR6_ scores were lower only than those of QU (Table 1). In the majority of tests, (−)-epicatechin (ECA) was found to be superior when the results were expressed per unit of weight (Figure 3), while the higher oligomers performed best with regard to molar activity (Table 1). The latter fact is in agreement with previous findings and is connected with the polyhydroxylated structures of oligomeric proanthocyanidins [20]. However, as ECA predominated in most of the examined extracts, particularly in DEF (Figure 2), it might be regarded as a good representative of the active flavanols of *S. domestica* leaves.

Some interesting differences between the assays were also observed regarding the relative activity of flavonols and flavanols. For example, flavonoids QU and QCT were particularly effective in OH^•^ scavenging, while flavan-3-ol derivatives were superior in terms of O_2_^•−^ quenching (Figure 3). This observation might be connected with the decisive role of the C2–C3 double bond of flavone derivatives in OH^•^ radical-antioxidant interactions [21]. Conversely, a saturated C2–C3 bond was reported to be favorable for O_2_^•−^ scavenging [22].

Chlorogenic acid (CHA), the sole representative of caffeoyl quinic derivatives, was also a highly active compound in most of the tests. However, it was found to be visibly inferior to the other tested compounds in the HClO quenching assay (Figure 3). This may be connected with the fact that in case of flavonoids and flavan-3-ol derivatives, the HClO scavenging occurs mainly via electrophilic reactions on the A and C rings of a flavonoid skeleton (Figure 1), whereas the *ortho*-dihydroxyphenyl group (catechol moiety) present in the investigated flavan (ring B) and caffeic acid derivatives does not undergo chlorination [23]. Nevertheless, the relatively high AAE_MR6_ and TE_MR6_ scores of CHA, comparable to those of QCT and procyanidin C1 (PC1), respectively (Table 1), as well as its abundant occurrence, especially in BF, WR and MED (Figure 2), make CHA an important determinant for the activity of *S. domestica* extracts.

### 2.2. Synergistic Effects in O_2_^•−^ Scavenging

The complex composition of natural products represents one of their main advantage in comparison to synthetic drugs. It enables, among other effects, possible synergistic effects between components that lower the concentration of the drug required for the desired outcome and the risk of adverse events. In the case of antioxidant activity, considerable synergy has been found for molecules with catechol moiety [24,25]. Similar effects might thus be anticipated between the main constituents of *S. domestica* leaves, as they all are catechol-type polyphenols.

To assess the possibility of such effects, the predicted extract activity indices (PEA) were determined based on the extracts’ phytochemical profile. For a given extract in a given assay, the PEA value was understood as the weighted sum of the markers’ activities in the assay (in AA equivalents), with the percentage content of the markers in the extract used as weights. Assuming the effects of the markers were purely, or in large part, additive, the PEA values should be good predictors of the relative capacities of the extracts in the assay, and the correlation coefficients between the PEA and the extract activity parameters should be high and statistically significant. Such relationships were found for most of the assays (Table 2). The exception was the O_2_^•−^ scavenging test, thus suggesting, in this case, the presence of possible pronounced synergy/antagonism effects. For a closer investigation, representatives of the three main classes of polyphenols present in *S. domestica* leaf (flavanols, flavonols, and quinic acid pseudodepsides), i.e., ECA, QCT, and CHA, were chosen.

**Figure 4:**
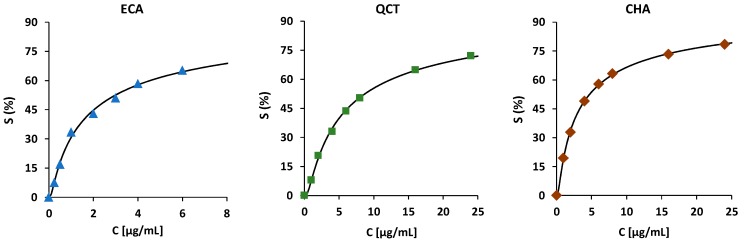
Observed mean data (*n* = 5) and the fitted dose-response curves for the main extracts’ constituents in the O_2_^•−^ scavenging capacity assay.

To calculate the combined effects of two or more compounds and estimate the degree of potential synergy, individual dose-response curves have to be estimated [26]. Therefore, the experimental data for each tested analyte was first fitted into different sigmoid functions, among which, the Weibull survival distribution equation offered the best fitting parameters (Figure 4, Table 3). This model has previously been found to be a good approximation of the antioxidant activity in different systems [27]. Then, because synergy depends on both the properties and the exact dose of each constituent [11,25,26], each combination of the compounds was tested in five concentration ratios. To evaluate the observed effects, the combination indices (*CI*) were calculated (Table 4). The *CI*s are indices based on the Loewe additivity model, which juxtaposes the concentrations of the analytes actually used in the assay with those theoretically required to produce the same effect (calculated from the individual dose-response curves) [28]. Consequently, a *CI* equal to 1 indicates additivity, while a *CI* less then or more than 1 indicates synergy or antagonism, respectively [28]. The concentrations and proportions in the experiments were chosen based on the individual compound scavenging efficiency (less than 60%), the relative proportions of the compounds in the extracts, as well as the range of physiological levels of plant-derived phenolic compounds in human plasma (1–5 µg/mL) available after oral administration [3,4].

As shown in Table 4, with the exception of the QCT–ECA in the proportions 2:1 and 4:1 µg/mL, and CHA–ECA in proportion 1:4 µg/mL, all other combinations showed some synergy effects, although with different intensities. The best effects were observed for QCT–CHA, which presented *CI* values significantly lower than 1, regardless of the concentration ratio. For example, QCT–CHA produced an approximate 50% scavenging effect in the proportion 1:2 µg/mL (total concentration of 3 µg/mL), while up to 4.19 µg/mL of pure CHA or 7.80 µg/mL of pure QCT was needed to achieve the same effect. Consequently, while CHA and QCT were separately less effective than ECA, their combination expressed similar efficacy. In addition, the scavenging potential of ECA might also be further increased by the presence of either of the two other compounds. Especially effective was the combination ECA–CHA; e.g., a quenching effect greater than 50% was achieved by only 2 µg/mL of 1:1 µg/mL ECA–CHA in comparison to 2.62 µg/mL of pure ECA. In light of the obtained results, the disturbed correlation between PEA and the extracts activity in the O_2_^•−^ scavenging assay might be explained by the differences in the extracts’ profiles, i.e., in the proportions between different groups of constituents. For example, phenolic rich DEF and EAF exhibited activities similar to those of BF, a relatively poor fraction in polyphenols but distinguished by a well-balanced composition [3]. In particular, the largest proportion of CHA was found with the ratios CHA:flavonoids and CHA:flavan-3-ols in an optimal range of about 3:1 and 1.5:1, respectively (Figure 2).

Due to the wide occurrence of the three investigated polyphenols, the obtained results might be applicable for other plant materials with a composition similar to that of *S. domestica*. For example, the extracts from the flowers of a related *Sorbus* species, *S. aucuparia*, have been also been proven to possess a relevant antioxidant activity [29]. However, despite a higher content of total phenolics, the activity of the *S. aucuparia* extracts in the O_2_^•−^-scavenging test was only comparable to or even lower than that of the extracts investigated in the present study. The largest difference was observed between BF fractions of the two species. Since, in contrast to *S. domestica*, *S. aucuparia* contains a very small amount of ECA and low-molecular-weight procyanidins, the discrepancies in activity might be due to the limited synergistic effects between these compounds and other polyphenols (flavonoids and caffeoylquinic acids). Apart from O_2_^•−^-quenching, the lower relative activity of *S. aucuparia* in comparison to *S. domestica* was also noticeable in other scavenging tests, with the exception of HClO-quenching. This observation suggests that in those cases, the synergic effects might still be present and worth investigation. Indeed, the interactions between flavonoids, flavan-3-ols and caffeic acid derivatives, although relatively rarely studied so far, have been revealed in different models of antioxidant activity [25,30,31]. For example, a study of synergistic activity between phenolic acids and flavonoids, found that CHA–QU (0.6 + 0.15 µmol/mL) and CHA–RT (0.6 + 0.15 µmol/mL) exhibit enhanced ferric reducing antioxidant power (FRAP) by 17.2% and 5.5%, respectively [25]. Similarly, a study of the constituents of green tea and *Osmanthus fragrans* flowers found the highest antioxidant effects (in DPPH assay) to be associated with a combination of acteoside (caffeoyl-rhamnosyl-glucoside of hydroxytyrosol) and ECA and caffeic acid and ECA [32]. In the same model, synergistic effects were demonstrated between CHA and simple phenolic acids [33], while in ORAC assay, CHA acted synergistically in two- to four-compound combinations with flavonoids from *Citrus sinensis* [31]. The interactions between the individual compounds and groups of compounds can further translate not only to the improved efficacy of a single plant material but also to enhanced activity of plant material combinations. For example, the antioxidant capacity of tea (*Camelia sinensis*) was improved in combined formulation with the *Vitis vinifera* seed (proanthocyanidins), *Punica granatum* and *Phyllanthus emblica* fruits (hydrolysable tannins), *Ginkgo biloba* leaf (flavonoids), and *Cinnamomum cassia* bark (condensed tannins) [34].

## 3. Materials and Methods

### 3.1. General

High-purity reagents and standards used for fluorometric and spectrophotometric assays including xanthine, xanthine oxidase from bovine milk, nitrotetrazolium blue chloride (NBT), horseradish peroxidase, hydrogen peroxide, 4-aminoantipyrine, phenol, iron (II) sulfate heptahydrate, salicylic acid, 5-thio-2-nitrobenzoic acid, 4,5-diaminofluorescein, Evans blue, sodium borohydride, ethylenediaminetetraacetic acid, diethylenetriaminepentaacetic acid, QU dihydrate, QCT, RT, CHA, TX, and AA were from Sigma-Aldrich (Seelze, Germany/St. Louis, MO, USA), phosphate-buffered saline (PBS) was from Biomed (Lublin, Poland), sodium nitroprusside and NaOCl were from Avantor Performance Materials (Gliwice, Poland), while ECA, PB2, and PC1 were purchased from Phytolab (Vestenbergsgreuth, Germany). The species-specific flavonoids HRQ and PRQ were isolated earlier in our laboratory from the leaves of *S. domestica* [4]. All other chemicals were of analytical grade and obtained from Avantor Performance Materials (Gliwice, Poland). All activity studies were performed using 96-well plates and microplate readers: SPECTROstar Nano (BMG LabTech, Ortenberg, Germany) and Synergy HTX (BioTek, Winooski, VT, USA).

### 3.2. Plant Material and Extracts Preparation

The analyses were performed using extracts (MED, DEF, EAF, BF, WR) obtained by a fractionated extraction of the leaves of *S. domestica* L. The plant material was collected in September 2015 in the Arboretum (51°49′N, 19°53′E), Forestry Experimental Station of Warsaw University of Life Science (SGGW) in Rogow (Poland) and authenticated by Piotr Banaszczak (Head of the Arboretum). The preparation of the extracts and their standardization were described previously [3].

### 3.3. Antioxidant Activity Measurements

The scavenging activity of the analytes towards multiple oxidants was evaluated using relevant spectrophotometric and fluorimetric methods. The O_2_^•−^ scavenging activity was determined according to Granica et al. [35] in a xanthine/xanthine oxidase system with NBT used for detection. The ability to scavenge H_2_O_2_ was evaluated according to Marchelak et al. [36] by monitoring the level of quinoneimine generated in the reaction of 4-aminoantipyrine, phenol and H_2_O_2_, catalyzed by horse radish peroxidase. The NO^•^ scavenging activity was measured acc. to Czerwińska et al. [37] using 4,5-diaminofluorescein as NO^•^ probe. The ability to scavenge HO^•^ was assayed using the method by Marchelak et al. [36] with the level of HO^•^ monitored in the presence of salicylic acid. The HClO scavenging effect were evaluated according to Czerwińska et al. [37], with 5-thio-2-nitrobenzoic acid used for detection. The ability to scavenge ONOO^–^ (obtained synthetically) was determined according to Krzyzanowska-Kowalczyk et al. [38], by measuring the inhibition of Evans Blue dye oxidation. The results were expressed as SC_50_ values (the concentration of the analyte that decreases the initial amount of the oxidant by 50%) and calculated from concentration-scavenging curves (5–10 calibration points). For direct comparison, the results were expressed in equivalents of AA (extract activity parameters, AAE) and TX (TE) per dry matter (µmol AA/mg dw and µmol TX/mg dw, respectively). Then the total antioxidant potential of each analyte was calculated with respect to both standards as a sum of the results from particular tests (AAE_MR6_ and TE_MR6_, respectively).

### 3.4. PEA Index Calculation

PEA indices, understood as the weighted sum of the markers’ activities in a particular extract, were calculated for each extract in each assay, according to Equation (1):

(1) $$\mathrm{PEA}=\sum c_{n}a_{n}$$

where *c_n_* is the relative content of compound *n* in the extract and *a_n_* is the activity of compound *n* in a particular assay expressed in AA equivalents.

### 3.5. Synergistic Effect Measurement

The synergistic effects between the representatives of the main extracts’ constituents (QCT, ECA, and CHA) were tested in an O_2_^•−^ scavenging capacity assay. First, the scavenging percentage (*S*) of the three individual compounds was tested in different concentrations (*c*) – 0.25–6 µg/mL for ECA and 1–24 µg/mL for QCT and CHA, and dose-response curves (eight calibration points) were obtained by fitting the experimental data into the Weibull function (Equation (2)) [39]:

(2) $$S\left(c\right)=Le^{-ln2{\left(\frac{c}{m}\right)}^{k}}$$

where *L* is the maximum possible response and was set to 100%, the parameter *m* corresponds to the concentration required for 50% radical quenching (SC_50_), and the parameter *k* is related to the maximal slope of the response (Figure 4, Table 3).

Then, the compound combinations (QCT–CHA, QCT–ECA, and CHA–ECA) were tested in different concentration ratios (4:1, 2:1, 1:1, 1:2, and 1:4) and the combination indices (*CI*) were calculated according to Equation (3):

(3) $$CI=\;\frac{c_{1}}{c_{x,1}}+\frac{c_{2}}{c_{x,2}}$$

where *c*_1_ and *c*_2_ are the concentrations of the compounds actually used in the assay, and *c_x_*_,1_ and *c_x_*_,2_ are the concentrations theoretically required (calculated from the dose-response curves) to produce the obtained effect *x*. A *CI* equal to 1 indicates additivity, while a *CI* less then or more than 1 indicates synergy and antagonism, respectively. For each dose combination, the experiment was run five times and a 95% confidence interval for each *CI* was obtained to evaluate its statistical significance.

### 3.6. Statistical Analysis

The results were reported as means ± *SD* (standard deviation) for the indicated number of experiments. The normality of the distribution of the results was verified using the Shapiro–Wilk test, and the homogeneity of variances was verified using Levene’s test. The significance of the differences between samples and controls was determined with a one-way ANOVA, followed by the post hoc Tukey’s test for multiple comparisons. The correlations were evaluated by calculating Pearson correlation coefficients. All calculations were performed using the Satistica13 Pl software for Windows (StatSoft Inc., Krakow, Poland). *p*-values less than 0.05 were regarded as significant.

## 4. Conclusions

This study is the first evaluation of the scavenging capacity of *S. domestica* leaf extracts and their activity markers towards in vivo-relevant oxidants. It demonstrated that both individual polyphenolic compounds and leaf extracts might be considered broad spectrum ROS/RNS scavengers and that ROS/RNS quenching might be one of the possible mechanisms of their protective effects against the oxidative/nitrative damage to human plasma noted in previous studies [3,4]. As demonstrated in the O_2_^•−^ scavenging test, some of the activity of the tested extracts might be a result of synergy between their constituents. This may be considered an advantageous feature and serve as a prompt for further studies of similar effects in more complex biological models. The observed relations between the three investigated phenolic groups might also be used for the interpretation of antioxidant effects in other plant materials of similar composition. However, more detailed research is required to identify other possible mechanisms and in vivo effects of the tested extracts. Non-direct mechanisms of antioxidant protection should first be verified, such as the influence of the analytes on gene expression and the activity of endogenous antioxidant enzymes, as well as the impact on ROS/RNS secretion at a cellular level.

## Figures and Tables

**Table 1:** Scavenging capacity towards multiple reactive oxygen/nitrogen species (ROS/RNS) in equivalents of standard antioxidants.

	AAE_MR6_		TE_MR6_			AAE_MR6_		TE_MR6_	
	μmol/mg	mol/mol	μmol/mg	mol/mol		μmol/mg	mol/mol	μmol/mg	mol/mol
**Extracts**					**Markers**				
MED	14.31	-	38.79	-	QU	72.24	21.82	145.33	43.89
DEF	42.37	-	102.16	-	QCT	49.06	21.98	116.09	52.01
EAF	47.36	-	106.71	-	HRQ	29.77	18.16	62.45	38.09
BF	31.20	-	84.82	-	PRQ	29.62	17.18	62.40	36.19
WR	4.21	-	11.62	-	RT	25.39	15.49	57.23	34.91
**Standards**					ECA	59.27	17.19	253.26	73.45
					PB2	55.68	32.18	189.75	109.68
AA	34.07	6.00	130.06	22.89	PC1	55.07	47.69	156.56	135.58
TX	97.10	24.28	23.97	6.0	CHA	45.69	16.17	154.12	54.56

AAE_MR6_ and TE_MR6_, total activity scores of the analytes—the sums of the activity parameters in all assays, expressed in micromolar equivalents of ascorbic acid (AA) and Trolox (TX) per mg of dry matter (values from Figure 3) and in molar equivalents of AA and TX per mol of the pure compounds.

**Table 2:** Predicted extract activity indices (PEA) values for extracts in particular assays and their correlations with extracts’ activity parameters (AAE).

PEA						
Extracts	O_2_^•−^	OH^•^	H_2_O_2_	HClO	NO^•^	ONOO^–^
MED	0.35	0.68	0.26	0.48	0.19	0.75
DEF	1.99	3.97	1.72	3.46	0.95	3.54
EAF	2.02	3.74	1.52	2.90	0.82	4.27
BF	0.45	0.93	0.32	0.52	0.31	0.99
WR	0.06	0.13	0.04	0.02	0.04	0.10
***r* (*p*)**						
**AAE**	0.8072 (0.099)	0.8794 (0.049) *	0.9043 (0.035) *	0.9227 (0.026) *	0.9779 (0.004) *	0.9035 (0.035) *

PEA, predicted extract activity index, understood as the weighted sum of the markers’ activities in AAE (ascorbic acid equivalents) with the percentage content of the markers in the extract used as weights, according to Equation (1); *r*, correlation coefficient between PEA values and the extracts’ activities in AAE for a particular assay; *p*, *p*-value for statistical significance of the correlation. The relationships marked with an asterisk are statistically significant at α = 0.05. * *p* < 0.05.

**Table 3:** Estimated model parameters for the dose-response curves of the main extracts’ constituents in the O_2_^•−^ scavenging capacity assay according to Equation (2).

Compound	Parameters		Model Significance			
	*m*	*k*	*SSE*	*R* ^2^	*F*-Test	*p*
ECA	2.622	−0.552	14.581	0.9964	2793.66	1.23 × 10^−9^
QCT	7.795	−0.638	5.580	0.9988	8296.13	4.72 × 10^−11^
CHA	4.192	−0.611	1.626	0.9997	41,930.01	3.66 × 10^−13^

Parameters: *m*, parameter corresponding to the concentration required for 50% radical quenching; *k*, parameter related to the maximal slope of response; *SSE*, the sum of squared errors of prediction; *R*^2^, coefficient of determination; *F*-test, value of the statistical Fisher variance ratio for the experimental data (the critical value at α = 0.01 is 11.259 for *n* = 8); *p*, significance level.

**Table 4:** The synergistic effects between the main extracts’ constituents.

Compound Combination	Concentration Ratio (µg/mL)	Theoretical Scavenging Efficacy (%)	Experimental Scavenging Efficacy (%)	CI ± 95% Conf.	Effect
QCT–ECA	4:1	47.75	47.71 ± 2.43	1.00 ± 0.10	additivity
	2:1	40.55	41.44 ± 2.22	0.97 ± 0.09	additivity
	1:1	35.95	39.82 ± 2.15	0.84 ± 0.08	synergy
	1:2	47.56	52.04 ± 2.86	0.81 ± 0.10	synergy
	1:4	59.12	61.57 ± 1.83	0.88 ± 0.08	synergy
QCT–CHA	4:1	43.68	54.77 ± 3.14	0.61 ± 0.08	synergy
	2:1	34.13	44.62 ± 3.02	0.64 ± 0.08	synergy
	1:1	27.46	37.44 ± 2.71	0.65 ± 0.07	synergy
	1:2	38.82	49.56 ± 3.86	0.62 ± 0.10	synergy
	1:4	51.61	61.04 ± 3.03	0.63 ± 0.09	synergy
CHA–ECA	4:1	55.91	60.39 ± 1.84	0.78 ± 0.07	synergy
	2:1	46.92	52.84 ± 2.66	0.75 ± 0.09	synergy
	1:1	40.12	51.82 ± 2.75	0.57 ± 0.07	synergy
	1:2	49.91	53.43 ± 2.63	0.84 ± 0.10	synergy
	1:4	60.26	60.33 ± 1.92	1.01 ± 0.10	additivity

Results are presented as mean values ± SD (*n* = 5) according to Equations (2) and (3). A *CI* (combination index) equal to 1 indicates additivity, while a *CI* less then or more than 1 indicates synergy and antagonism, respectively.

## Supplementary Materials

The Supplementary Materials are available online.
