# Supplementary material for: Sorbus domestica Leaf Extracts and Their Activity Markers: Antioxidant Potential and Synergy Effects in Scavenging Assays of Multiple Oxidants
Source: Molecules. 2019 Jun 20;24(12):2289. doi: 10.3390/molecules24122289 (PMC6630621; doi:10.3390/molecules24122289)
Supplement: Supplementary file 1 [file molecules-24-02289-s001.pdf]

molecules

MDPI

Article Supplementary Materials:

Sorbus domestica leaf extracts and their activity markers: Antioxidant potential and synergy effects in scavenging assays of multiple oxidants

Magdalena Rutkowska[1*], Monika Anna Olszewska[1], Joanna Kolodziejczyk-Czepas[2], Pawel Nowak[2], Aleksandra Owczarek[1]

[1]Department of Pharmacognosy, Faculty of Pharmacy, Medical University of Lodz, ul. Muszynskiego 1, 90-151 Lodz, Poland; magdalena.rutkowska@umed.lodz.pl (M.R.); monika.olszewska@umed.lodz.pl (M.A.O.); aleksandra.owczarek@umed.lodz.pl (A.O.)

[2]Department of General Biochemistry, Faculty of Biology and Environmental Protection, University of Lodz, ul. Pomorska 141/143, 90-236 Lodz, Poland; joanna.kolodziejczyk@biol.uni.lodz.pl (J.K.-C.); pawel.nowak@biol.uni.lodz.pl (P.N.)

* Correspondence: magdalena.rutkowska@umed.lodz.pl (M.R.)

Supplementary Materials:

- **Figure S1.**

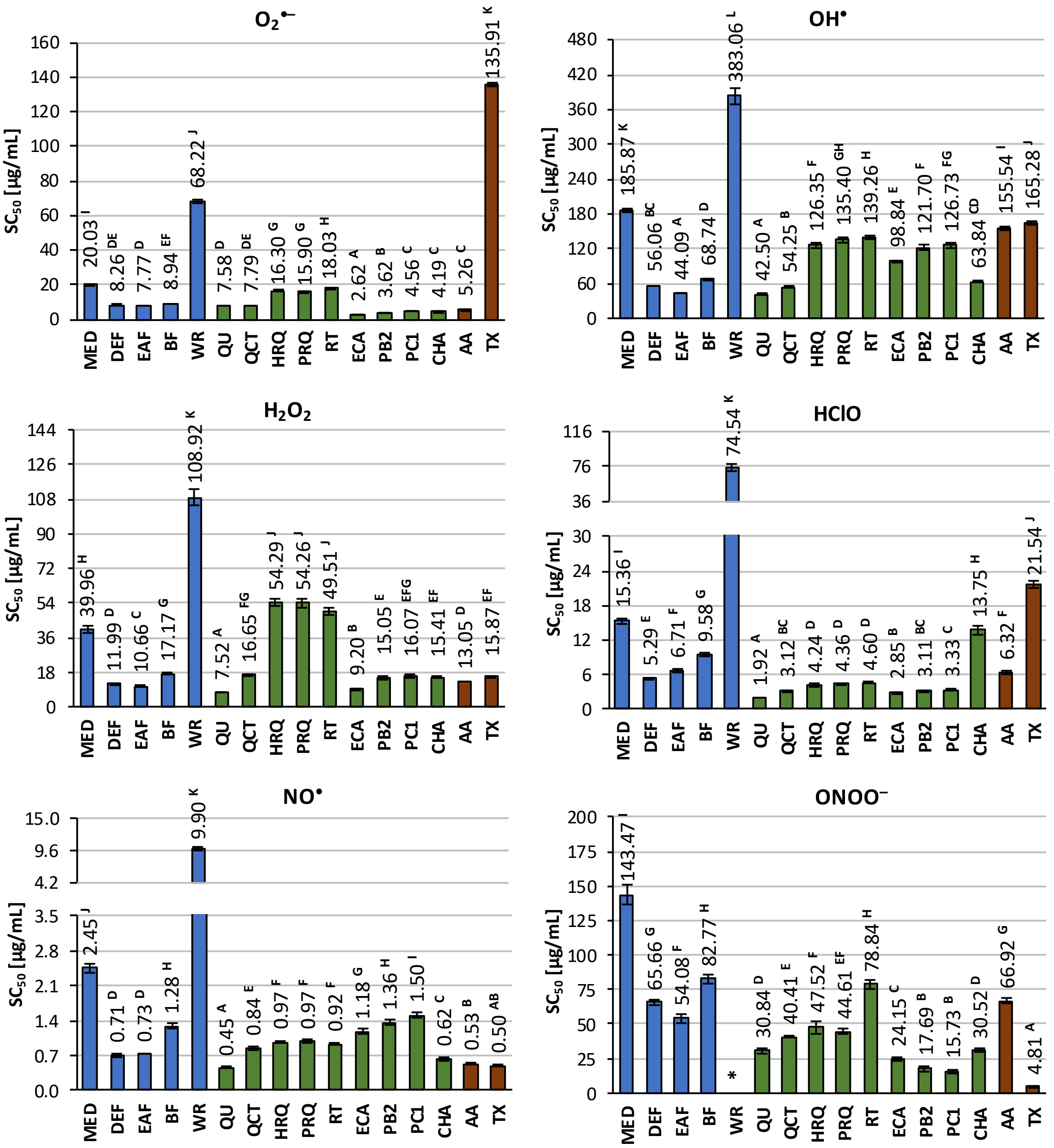

Figure S1. Antioxidant activity of *S. domestica* leaf extracts, their activity markers and standards towards *in vivo*-relevant oxidants. Results are presented as mean values of SC_{50} (amount of antioxidant needed to decrease the oxidant level by 50%) ± SD ($n = 3$), expressed in µg of the dry extract or standard/mL. For each parameter different letters A-K indicate significant differences ($p < 0.05$). The analyte marked with an asterisk is inactive in concentration up to 200 µg/mL. MED, methanol-water (7:3, *v*/*v*) extract; DEF, diethyl ether fraction; EAF, ethyl acetate fraction; BF, *n*-butanol fraction; WR, water residue; QU, quercetin; QCT, quercitrin (quercetin 3-*O*-α-L-rhamnopyranoside); HRQ, quercetin 3-*O*-(2''-*O*-β-D-glucopyranosyl)-α-L-rhamnopyranoside; PRQ, quercetin 3-*O*-(2''-*O*-β-D-xylopyranosyl) -α-L-rhamnopyranoside; RT, rutin; ECA, (−)-epicatechin; PB2, procyanidin B2; PC1, procyanidin C1; CHA, chlorogenic acid (5-*O*-caffeoylquinic acid); AA, ascorbic acid; TX, Trolox.
